# Experimental Research on the Supply of Working Fluid for Fixed Diamond Wire Slicing Based on Ultrasonic Capillary Effect

**DOI:** 10.3390/mi15070910

**Published:** 2024-07-13

**Authors:** Junying Zhao, Luqi Shen, Chunwei Zhang, Yanqing Wang

**Affiliations:** 1 Mechanical and Electrical Engineering, Luoyang Polytechnic, Luoyang 471942, China; 2 College of Mechanical and Vehicle Engineering, Taiyuan University of Technology, Taiyuan 030024, China

**Keywords:** fixed diamond wire slicing, photovoltaic silicon wafer, ultrasonic wave, capillary effect

## Abstract

Thin wafers and thin wires are beneficial to the photovoltaic industry for reducing costs, increasing efficiency, and reducing the cost of electricity generation. It is a development trend in solar silicon wafer cutting. Thin wire cutting reduces the kerf between silicon wafers to less than 50 μm. Therefore, it is extremely difficult to supply cutting fluid to the cutting area. And this affects cutting performance. This paper proposes the use of the capillary effect produced by ultrasonic waves in fixed diamond wire slicing to improve the cutting fluid supply and reduce wafer adsorption. To explore the rules of ultrasonic capillary action between two plates and guide the industrial applications, the effects of the distance between parallel plates, the distance from the bottom of the parallel plates to the ultrasonic radiation surface, the non-parallelism between the plates, the temperature of the working fluid, the ultrasonic action time, and the type of working fluid on the liquid level rise height were studied. The conclusions can be used to guide the improvement of the supply of working fluid in fixed diamond wire slicing.

## 1. Introduction

With the development of the solar industry, obtaining higher-efficiency cell performance and reducing wafer costs have become the main development trends. Fixed diamond wire slicing is widely used to machine hard and brittle material, such as mono-crystalline silicon, polycrystalline silicon, sapphire, silicon carbide, and so on [1]. To decrease wafer costs, reducing wafer thickness and kerf loss are the two most effective ways. Kerf loss is determined by the wire diameter used in fixed diamond wire slicing. The diamond wire core used in this study is around 40 µm, with the trend moving to less than 30 µm within the next five years [2,3,4,5]. The use of such a thin wire introduces many key technical challenges, such as a smaller breaking load and lower cutting fluid carrying capacity. The latest research focuses on model building [6,7] and the machining performance of fixed diamond wire slicing [8,9]. As a machining factor, the supply of working fluid and its effects on machining performance has been studied less.

In fixed diamond wire slicing, the cutting fluid is used to moisten the wire, to clean the Si particles generated by cutting, to reduce the resistance of the heat generated from the workpiece cutting, and to cool the workpiece. This can extend the service life of the wire, improve the cutting rate effectively, prevent the workpiece from warping, and achieve the best cutting quality. So, to supply the cutting fluid to the cutting area efficiently, a machining strategy to increase wire speed is proposed. Diamond grains are distributed on the surface of the wire core. Under the action of high wire speed, the cutting fluid is carried into the middle part of the silicon brick. The maximum wire speed used today on newly developed diamond wire saw machines is over 40 m/s. Further, increasing the wire speed poses more challenges [10]. 

Wire electrical discharge machining is a process that works by continuously feeding a wire electrode under tension. Fluid flow in the kerf machined by wire electrical discharge machining and the fixed diamond wire slicing has many similar characteristics. In multi-wire electrical discharge machining, by increasing the wire running speed, a higher-speed machining fluid flow can be formed around the wire [11]. In addition, a new jet flushing method in which the nozzle is tilted to the wire running direction was investigated; it was found that the tilted nozzle could decrease the debris stagnation area, and the removal rate could be improved [12,13]. Similar measures are also adopted by the fixed diamond wire slicing. Qiu proposed an improved cooling and lubrication method using a water tank bath for diamond wire sawing [14]. The capillary penetration mechanism of the cutting fluid supplied by electrostatic spraying in diamond wire sawing was researched [15]. In addition, the application of nano-silicon carbide cutting fluids in polysilicon cutting was researched to improve the lubrication performance [16,17]. However, methods to further improve the supply of working fluid during machining still need to be explored.

The United States patent application publication No. 20120167733A1 discloses a cooling device to improve the cutting capability and efficiency of the fixed diamond wire slicing system [18]. The Chinese patent application publication No. CN 103085179 and the European patent application No. EP 3 292 968 A1 disclose a fixed diamond wire slicing apparatus [19,20]. An ultrasonic vibrating plate is mounted in the tank. With the slicing of the Si brick, it is gradually immersed in the cutting fluid. Each of the cut grooves has a width that allows the cutting liquid to enter the cut grooves by capillary action. Under the action of ultrasonic vibration, the capillary effect is enhanced, and its ability to supply cutting fluids to the processing area is improved. Scientifically utilizing the capillary effect under ultrasonic vibration is an important research topic. However, few studies have investigated this topic. 

**Figure 1:**
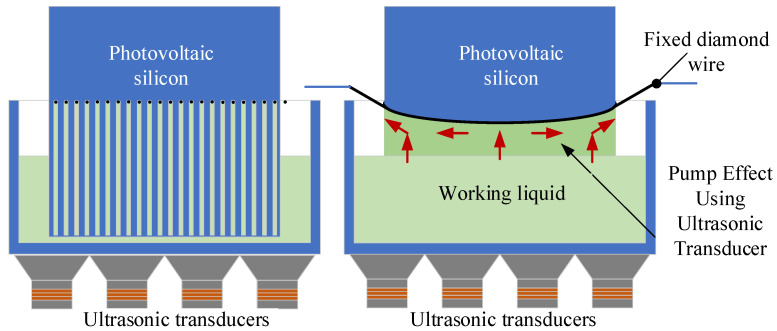
Suppling of working fluid based on the capillary phenomenon.

Based on the capillary phenomenon in the narrow slit, ultrasonic vibration is introduced to the slicing process, as shown in Figure 1, thereby increasing the height of the liquid rise in the narrow kerf, thus improving the cooling and lubrication conditions during slicing. Due to the non-transparency of the silicon wafers, it is difficult to observe the liquid level in the kerf. Therefore, the purpose of this paper is to build transparent experimental conditions for observable rises in liquid height and then study the effects of ultrasound on capillary phenomena under the conditions of large-size silicon wafers and ultra-fine wire cutting, and to obtain the basic laws of the capillary effect, to guide further industrial applications.

The novelty of this work is the focus on a new working fluid supply method based on the ultrasonic capillary effect for fixed diamond wire slicing, ensuring that working fluid penetrates the tool-to-workpiece point of contact, and thus impacts the material removal process. This study aims to investigate the impact of the main process parameters on the liquid rise height of the new working fluid supply method and to clarify the criteria for selecting parameters. The study includes the following steps. The first is to build an experimental setup and the measuring method of the liquid rise height. In the experimental section, influencing parameters, such as the kerf width, the distance between the ultrasonic radiation surface and the bottom of the kerf, the non-parallelism of the kerf, the cutting fluid temperature of the surfactants, and the ultrasonic action time on the liquid rise height, were studied. This study provides basic data support for the industrial application of this new working fluid supply method.

## 2. Experimental Setup

**Figure 2:**
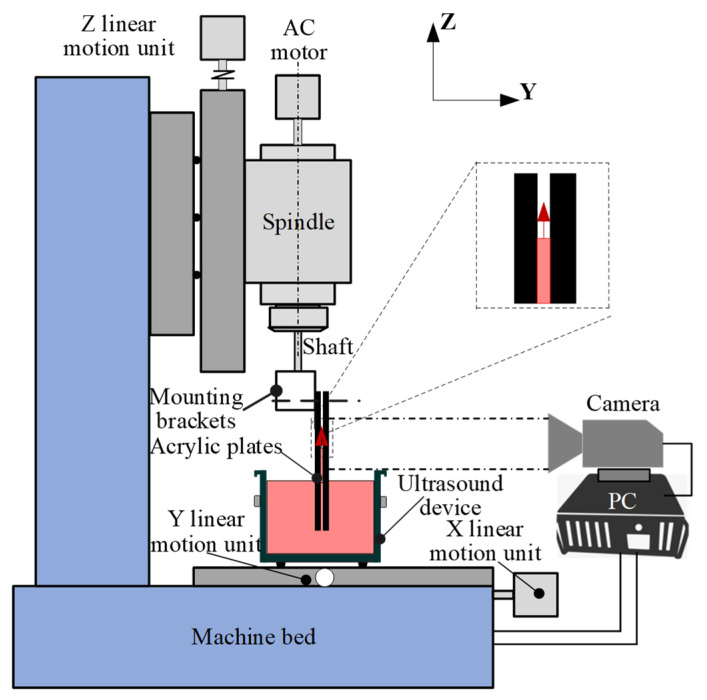
Schematic diagram of the experimental setup.

The experimental setup is shown in Figure 2. To facilitate the observation of experimental phenomena, the experimental setup uses two transparent acrylic plates to simulate the kerf. The size of the acrylic plate is 300 mm × 200 mm × 5 mm. The distance between the two parallel plates is adjusted by changing the feeler gauge inserted into the middle of the acrylic plates. A special mounting bracket is designed for installing the two acrylic plates. The designed experimental device is installed on the Z-axis pallet of a three-axis CNC machining center (Shenyang Machine Tool Co., Ltd, Shenyang, China. BRIO Miller 8); the main technical parameters are listed in Table 1. By changing the position of the Z-axis table with a positioning accuracy of 5 μm, the distance between the bottom of the two acrylic plates and the ultrasonic radiation surface is changed. 

The main parameters of the ultrasonic device are listed in Table 2.

**Figure 3:**
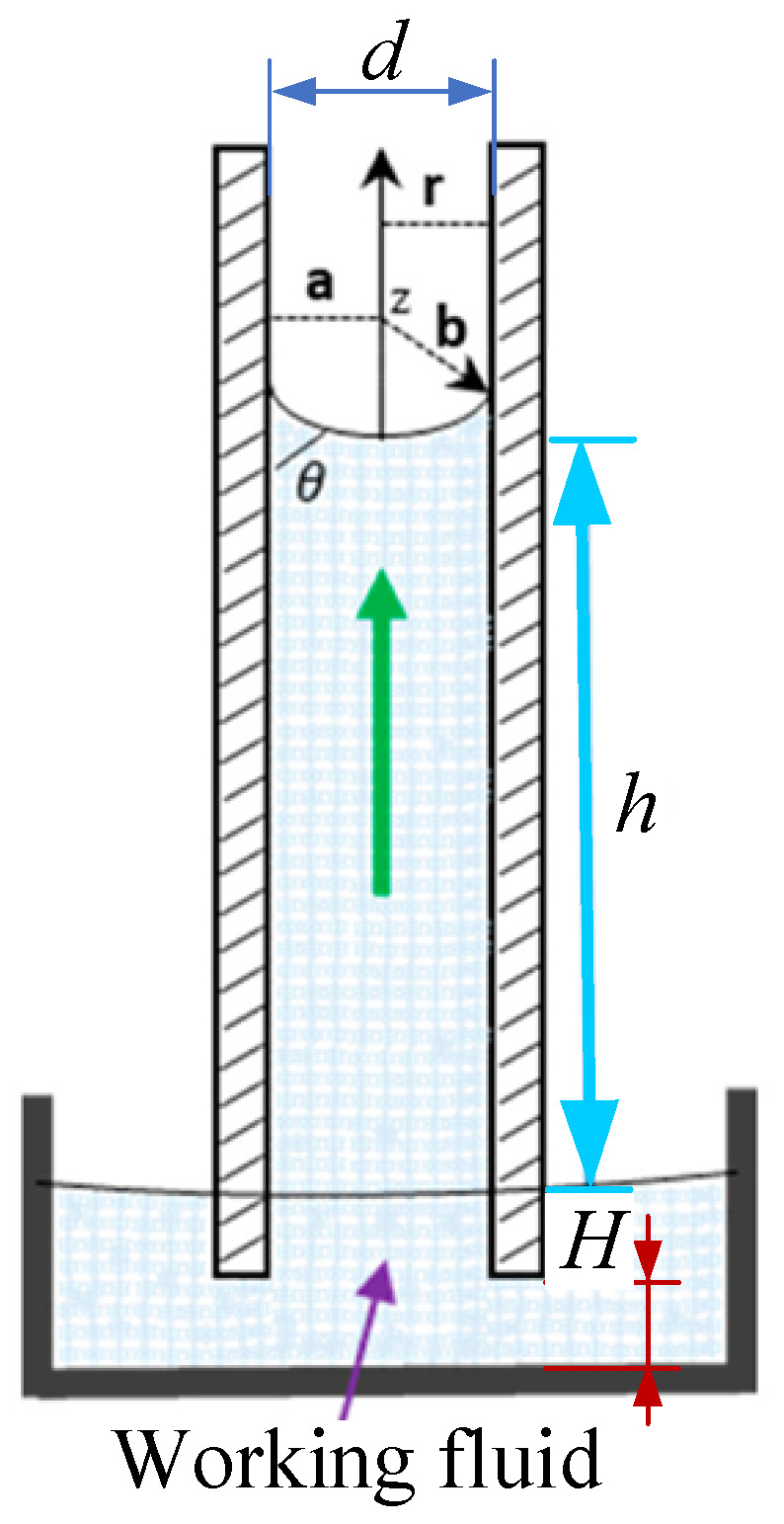
Schematic diagram of the liquid level rise height.

The method for measuring the liquid rise height is as follows: paste flexible scales with 1 mm resolution are glued on the two acrylic plates to record the liquid rise height *h*. To facilitate the observation of the liquid level position, 1% red pigment by volume rate was added to the working fluid. Distance from the bottom of the parallel plates to the ultrasonic radiation surface is marked as *H*, as shown in Figure 3. 

The experimental parameters used in this paper are listed in Table 3.

## 3. Results and Discussion

### 3.1. Effects of the Parallel Plate Spacing d

The height of the liquid rise without ultrasonic action was measured. Based on the experiment, the height of the liquid rise between two parallel plates is in the range of 4 mm to 6 mm, and the average value of 5 mm is used as the comparison group result.

**Figure 4:**
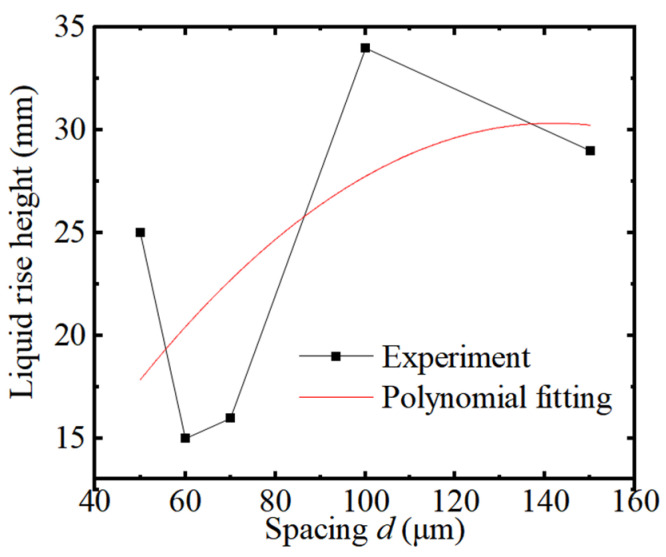
The liquid rise height vs. *d.*

The variation of the liquid rise height vs. the parallel plate spacing *d* is shown in Figure 4. It can be seen from Figure 4 that the liquid rise height first increases, and then decreases as the spacing *d* increases, and reaches the maximum rise height at 100 μm spacing *d*.

The spacing *d* affects the pressure between the capillary plate, which causes the liquid to rise. When the spacing *d* is less than 0.10 mm, the liquid rise height is further improved, which is mainly caused by ultrasonic cavitation. The collapse of cavitation bubbles leads to an increase in the liquid pressure, thus promoting the rise in liquid. The smaller spacing *d* leads to a reduction in cavitation bubbles, that is, the effective occurrence area is reduced. The flushing pressure decreases, thereby causing the liquid level to decrease. It was further found that the liquid rise height after adding ultrasonic vibration is between 15 and 35 mm, which is higher than the corresponding liquid rise height of 5 mm without ultrasonic vibration action. It was proven that the liquid rise height further increases after adding ultrasonic vibration action, which helps to strengthen the ultrasonic capillary effect.

### 3.2. Effect of the H 

**Figure 5:**
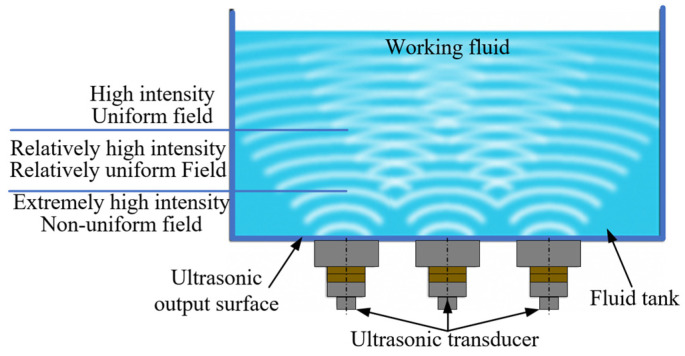
Schematic diagram of ultrasonic intensity distribution.

The distance between the bottom end of the two parallel plates and the ultrasonic radiating surface of the water tank is expressed as H in this paper. It can be adjusted with 1 μm resolution by the Z-axis of the machine tool. Figure 5 shows the effects of *H* on the height of the liquid rise. It can be seen from the figure that with the increase in *H* in the range of 2 mm~32 mm, the height of the liquid rise increases with *H*. And the height of the liquid rise reaches the maximum value at 32 mm *H*. This is because at this distance, a relatively high intensity and relatively uniform field is generated. The cavitation bubbles are generated more fully, and thus, a larger cavitation pressure is generated, which helps to increase the liquid rise height.

**Figure 6:**
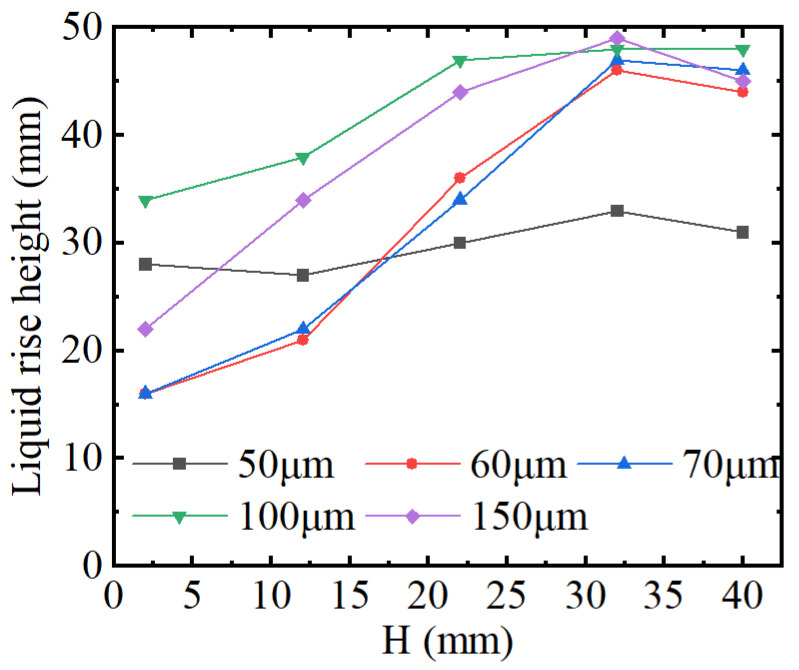
The effects of the H on the liquid rise height.

When *H* is in the range of 32 mm~40 mm, the rising height of the liquid level shows a decreasing trend, as shown in Figure 6. When *H* is greater than 40 mm, the rising height of the liquid level decreases. This is because the pressure caused by the collapse of the cavitation bubbles decreases. Therefore, the distance *H* in engineering applications should be optimally chosen and a better value of 32 mm can be used.

### 3.3. Effect of Non-Parallelism between Two Plates

**Figure 7:**
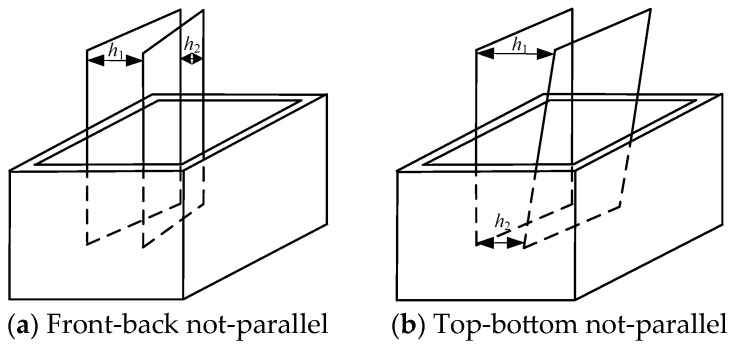
Schematic diagram of non-parallelism.

The effects of non-parallelism between two sides of two plates were investigated. The schematic diagram of non-parallelism between two sides of plates in this paper is shown in Figure 7. The non-parallelism was adjusted by inserting feeler gauges of different thicknesses. 

#### 3.3.1. Front-Back Sides Not-Parallelism

The parameters of the front-back side spacing are listed in Table 4.

**Figure 8:**
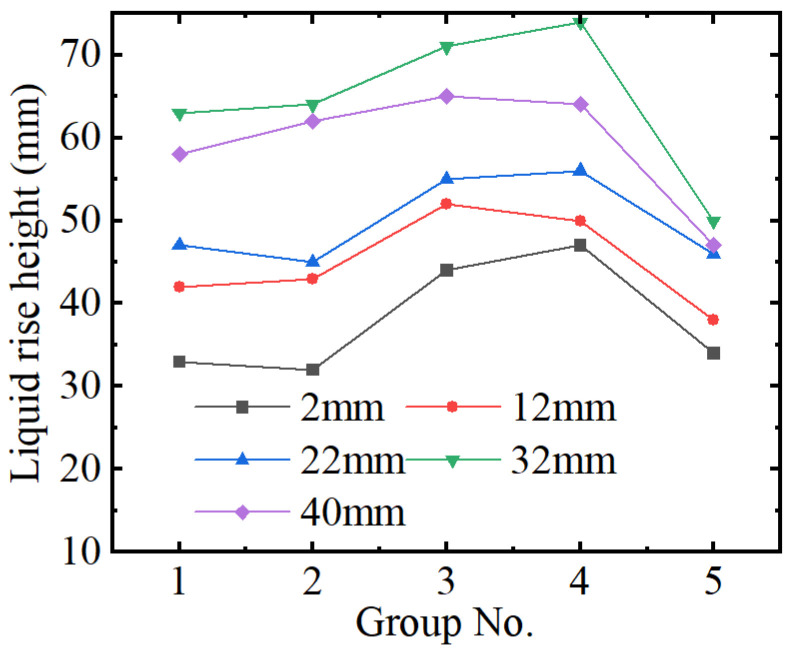
The height of the liquid rise (front–back non-parallelism).

Figure 8 shows the effects of the front–back non-parallelism on the height of the liquid rise. It can be seen from Figure 8 that the liquid level reaches its peak value in Group 3 (F: 0.30, B: 0.10). In addition, the height of the liquid level rise in Group 3 (F: 0.50, B: 0.10) and Group 4 (F: 0.30, B: 0.10) is significantly greater than that in Group 5 (F: 0.10, B: 0.10), that is, two parallel plates. It can also be seen from Figure 8 that the larger the distance between the plates on one side, the more obvious the rise and fall of the liquid height. The height of the liquid rise reaches the maximum value at 0.30 mm on the front and 0.10 mm on the back.

#### 3.3.2. Up-Down Sides Not-Parallelism

The parameters of the up-down side spacing are listed in Table 5.

**Figure 9:**
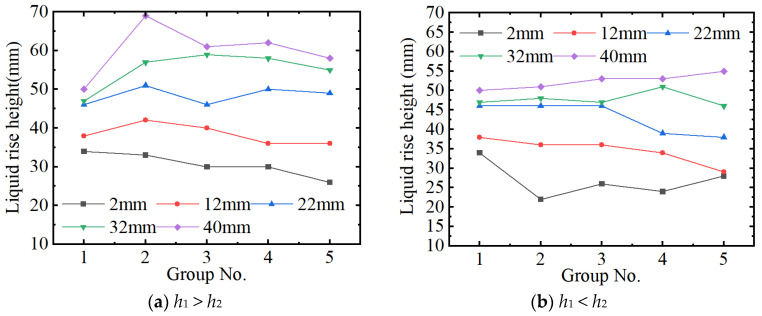
The height of the liquid rises (up-down non-parallelism).

Figure 9 shows the experiment results for the height of liquid rise of *h*_1_ > *h*_2_ and *h*_1_ < *h*_2_. It can be seen from Figure 9a that the height of the liquid rise of two parallel plates is lower than that of two non-parallel plates (*h*_1_ > *h*_2_) for H 22 mm, 32 mm, and 40 mm. For H 2 mm and 12 mm, an opposite rule is shown. In addition, for Group 2 (Up: 0.20, Down: 0.10), the maximum height of liquid rise is obtained. 

It can be seen from Figure 9b that the height of the liquid rise of two parallel plates is higher than that of two non-parallel plates (*h*_1_ < *h*_2_) for H 2 mm, 12 mm, and 22 mm. It also can be seen from this figure that with the increase in the H_2_–H_1,_ the height of liquid rise decreases. But for the H 40 mm, an increasing trend is shown.

### 3.4. Effects of the Working Fluid

The surface tension of the liquid is a parameter affecting capillary action. So, experiments to evaluate the effect of liquid surface tension on the capillary rise dynamics were conducted. Four types of surfactants shown in Table 6 were used in experiments to change the surface tension of liquids. The classifications of surfactants are based on the composition of the polarity of the head group: nonionic, anionic, cationic, and amphoteric. 

**Figure 10:**
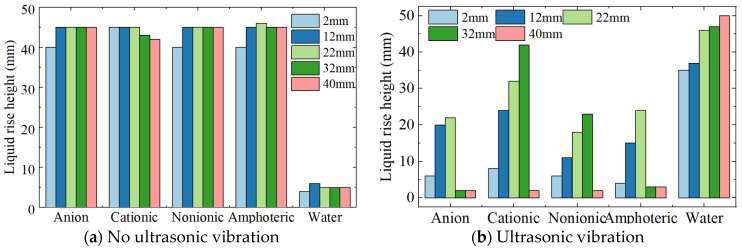
Liquid rise height vs. surfactants.

After adding surfactant to the working fluid, the liquid rising height obtained is shown in Figure 10. It can be seen from Figure 10a that when surfactant is added, the liquid height between parallel plates increases significantly even without ultrasonic action. In the water, the liquid’s rising height is about 5 mm. After adding surfactant, the rising height is between 40 mm and 45 mm. Therefore, the surfactant has a significant strengthening effect on the liquid’s rising height.

It can be seen from Figure 10b that with the ultrasonic action, the liquid rise height obtained using the cationic surfactant is the highest, followed by anionic and nonionic surfactants, whose rising heights are almost the same. The use of amphoteric surfactants has the smallest rising height.

The surface tension coefficient affects the pressure, which in turn affects the liquid’s rising height. Therefore, in silicon wafer slicing using fixed diamond wire slicing (DWS), cationic surfactants can be added to the working fluid to increase the liquid rise height, thereby improving the supply of working fluid in the slicing area.

### 3.5. Effect of Working Fluid Temperature

**Figure 11:**
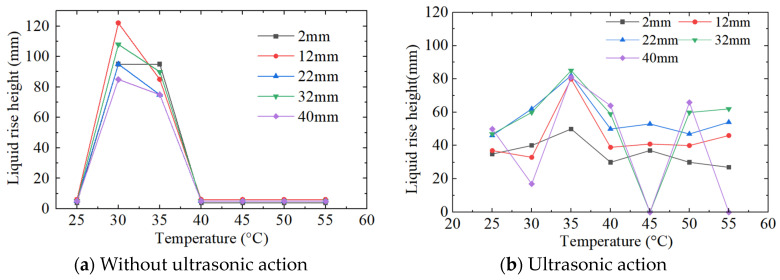
The effects of the working liquid temperature on the liquid rise height.

Figure 11 shows the effects of the working liquid temperature on the liquid rise height. It can be seen from Figure 11a that without the ultrasound action, the liquid height between parallel plates has a larger value at 30 °C and 35 °C; at 25 °C and in the range of 40~55 °C, the liquid rise height is small, and is maintained at about 5 mm; in the temperature range of 30~35 °C, the rising height of the liquid is between 60 and 120 mm, and significantly increases.

It can be seen from Figure 11b that after adding the ultrasonic action, the liquid height rises sharply and reaches the maximum value at 30 °C. In the range of 30 °C to 35 °C, the liquid rising height is significantly greater than that under other temperature conditions. In the range of 30–40 °C, the liquid height in the parallel plate decreases rapidly as the temperature rises. When the temperature is greater than 40 °C, the liquid height decreases slowly with the increase of the temperature. Within the temperature range used in this paper, the liquid height rises significantly in the range of 25–30 °C. In the range of 30~55 °C, there is a negative correlation between temperature and ultrasonic action.

The working fluid temperature affects the pressure in the capillary tube which causes the rise in liquid height. Therefore, it affects the rising height of the working liquid. According to the ultrasonic cavitation theory, the rise in the water temperature causes the saturated vapour pressure to rise rapidly. Although the bubble radius increases, the collapse time also extends significantly, which leads to a decrease in the bubble cavitation energy and the additional pressure. At the same time, the cavitation threshold of bubbles in the cavitation zone is increased, resulting in a larger drop in the liquid rise height between 30 °C and 40 °C.

In the slicing of photovoltaic silicon wafers using DWS, the working fluid is supplied at about 20~25 °C. Considering the slicing heat, the actual temperature in the processing area is higher than 20~25 °C. The experiment results show that with the ultrasonic action, even if the temperature rises, the liquid rise height is better obtained, which is beneficial to improving the liquid supply and lubrication during slicing.

### 3.6. Effect of Ultrasonic Vibration Time

**Figure 12:**
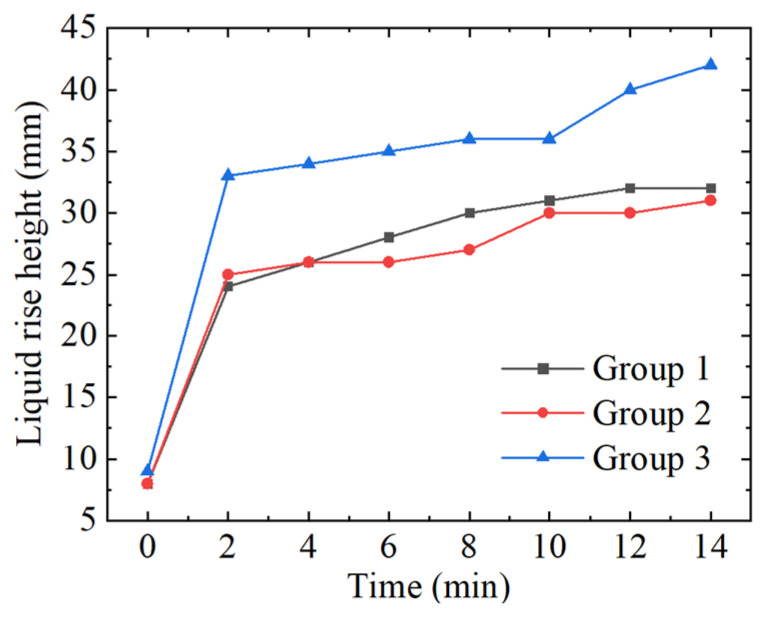
The change curve of the liquid rise height with the ultrasonic action time.

The curve of the liquid rise height with the ultrasonic action time is shown in Figure 12. It can be seen from Figure 9 that the height of liquid rise has the largest increasing rate in the first 2 min, and then decreases rapidly. Compared with the first and the second groups, the liquid rise height in the third group becomes higher. This is because with the increase in the ultrasonic time, the temperature of the working liquid increases and the liquid rise height becomes higher.

The slicing speed of photovoltaic silicon wafers is within the range of 1.5 mm/min~2 mm/min using DWS. Evidently, the rising speed of the working fluid is greater than the slicing speed. Therefore, in engineering applications, the application time of ultrasonic action can be synchronized with the cutting start time or slightly later than the cutting start time.

## 4. Conclusions

In fixed diamond wire slicing, improving the supply of working fluid within the slicing slit is of great significance. The effects of six parameters on the liquid rise height were revealed by experimental research. The main conclusions are as follows:

- With the help of ultrasonic vibration, a higher liquid rise height can be achieved by properly selecting parameters, which is a new strategy that helps improve liquid supply in fixed diamond wire slicing.
- With the decrease in the spacing *d* (slitting width), the cavitation bubbles in the parallel plates decrease and the liquid rise height decreases. The liquid rise height reaches the maximum value with a spacing *d* of 100 μm.
- Within the range of 2~32 mm H, the liquid rise height increases with the increase in H. The liquid rising height reaches the maximum value when H is 32 mm. When the H is greater than 32 mm, the liquid rising height decreases.
- After adding surfactant, the liquid rise height increased greatly even without ultrasonic action. The liquid rise height using the cationic surfactant reaches the maximum value, the anionic and nonionic surfactants have approximately equal liquid rise height, and the amphoteric surfactant has a lower rise height.
- The liquid rising height increases with the temperature rise in the range of 25~30 °C and reaches the maximum value at 30 °C. The liquid rising height decreases with the rise in fluid temperature in the range of 45~55 °C.
- Under the ultrasonic vibration, the liquid rising rate is the largest in the first two minutes and reaches a lower rate from 2 to 10 min. After 10 to 14 min, the liquid rising rate approaches zero and maintains a stable rising height.

The experimental data and results of this paper provide theoretical guidance for engineering applications and help propose new strategies to improve the supply of working fluid for fixed diamond wire slicing.

## Figures and Tables

**Table 1:** Technical parameters of Miller 8.

Technical Parameters	Value
Stroke	800 mm × 500 mm × 300 mm
Positioning accuracy	5 μm
Repeat positioning accuracy	3 μm
Maximum feed speed	800 mm/s

**Table 2:** Parameters of ultrasonic device.

Parameter	Value
Overall dimensions/mm	325 × 265 × 280
Water tank size/mm	300 × 240 × 150
Power supply	AC220–240 V 50 Hz
Ultrasonic power/W	240
Heating power/W	200
Ultrasonic frequency/kHz	40
Maximum capacity/L	10

**Table 3:** Experimental parameters.

Parameters	Value
*H*	2, 4, 6, 8, 10, 12, 22, 32, 40
Fluid temperature/°C	25, 30, 35, 40, 45, 50, 55
Ultrasonic vibration time/min	2, 4, 6, 8, 10, 12
Liquid pigment vol%	1%
Working fluid	Water
Spacing *d*/μm	50, 60, 70, 100, 150

**Table 4:** Spacing of the front-back sides.

Group		1	2	3	4	5
Spacing (μm)	Front	1000	750	500	300	100
	Back	100	100	100	100	100

**Table 5:** Parameters of the up-down sides’ spacing.

Unit (μm)	$h_{1}>h_{2}$		$h_{1}<h_{2}$	
	$h_{1}$	$h_{2}$	$h_{1}$	$h_{2}$
Group 1	100	100	100	100
Group 2	200	100	100	200
Group 3	300	100	100	300
Group 4	400	100	100	400
Group 5	500	100	100	500

**Table 6:** Surfactants.

	Group 1	Group 2	Group 3	Group 4	Group 5
Surfactant	Anion	Cationic	Nonionic	Amphoteric	/

## Data Availability

The data supporting the reported results have been completely declared in this article.
